# The prevalence of *Helicobacter pylori* infection and other risk factors among Mongolian dyspeptic patients who have a high incidence and mortality rate of gastric cancer

**DOI:** 10.1186/s13099-018-0240-2

**Published:** 2018-04-04

**Authors:** Oyuntsetseg Khasag, Gantuya Boldbaatar, Tserentogtoh Tegshee, Davaadorj Duger, Azzaya Dashdorj, Tomohisa Uchida, Takeshi Matsuhisa, Yoshio Yamaoka

**Affiliations:** 1 Mongolian National University of Medical Sciences, Ulaanbaatar, Mongolia; 2 Department of Environmental and Preventive Medicine, Oita University Faculty of Medicine, 1-1 Idaigaoka, Hasama-machi, Yufu, Oita 879-5593 Japan; 3 Department of Molecular Pathology, Oita University of Medicine, Yufu, Japan; 4 Department of Gastroenterology, Tama-Nagayama University Hospital, Nippon Medical School, Tokyo, Japan; 5 Department of Gastroenterology and Hepatology, Baylor College of Medicine and Michael De Bakey Veterans Affairs Medical Center, Houston, TX 77030 USA

**Keywords:** *Helicobacter pylori*, Risk factors, Gastric diseases, Mongolia

## Abstract

**Background:**

Mongolia has not only the second highest incidence rate but also the highest mortality rate for gastric cancer globally. In addition to gastric cancer, ulcerative disease complications are also life threatening; thus, investigating *Helicobacter pylori* infection and other risk factors is essential.

**Results:**

*H.pylori* infection was high in tested dyspeptic patients from all parts of Mongolia, with an overall infection rate of 80.0%. Logistic regression analysis showed that *H. pylori* infection was associated with gastritis (odds ratio; 9.0 ([95% confidence interval 5.0–16.2]); *p *< 0.0001). *H. pylori* infection (3.3 [2.0–5.4]; *p *< 0.0001) and > 40 years old (1.5 [1.0–2.0]; *p *< 0.02) were both associated with atrophy. However, > 40 years old (3.8 [2.4–6.0]; *p *< 0.0001) and high salt intakes (1.6 [1.0–2.3]; *p *< 0.02), but not *H. pylori* infection, were associated with intestinal metaplasia. Excessive amount of salt usage was dramatically higher in northern and western parts of Mongolia, where precancerous diseases, such as erosive esophagitis (for cardia cancer), severe atrophy, and intestinal metaplasia (for non-cardia cancer), were highly prevalent.

**Conclusions:**

*H. pylori* infection was the major gastric health problem among the Mongolian population. In addition, environmental factors such as high salt intake worsened the clinical outcome. Therefore, a nationwide screening and eradication of *H. pylori* infection as well as salt-reducing measures should be implemented.

## Background

Mongolia has the second highest incidence of gastric cancer (GC) worldwide [1]. According to the national health indicator data, digestive system diseases are the second most common disease among the Mongolian population [2]. Moreover, ulcerative disease complications such as bleeding and perforation are severe and life threatening [3, 4]. In 1994, *Helicobacter pylori* was declared to be a class I carcinogen [5, 6]. Currently, it is well known as the main etiological factor for chronic gastritis, peptic ulcer disease (PUD), mucosa-associated lymphoid tissue (MALT) lymphoma and GC [7, 8]. In addition, the infection was also reported as a risk factor for gastric dyspepsia in the Maastricht V guideline [9]. *H. pylori* is a flagellated, spiral-shaped, Gram-negative, microaerophilic bacteria which can survive in acidic environment and colonizes the gastric mucosa in half of the world population [10]. A current global systematic review showed that *H. pylori* infection continues to be a major public health issue worldwide, and approximately 4.4 billion individuals were estimated to be infected [10]. The prevalence of *H. pylori* infection varies geographically, based on ethnicity, age, socioeconomic factors, and is more frequent in developing than developed countries [11]. It has been attributed to low socioeconomic status, overcrowded condition, and inadequate hygiene [12, 13]. The infection is usually acquired in early childhood and reaches 80–100% during adolescence in developing countries [8] probably due to oral–oral, fecal–oral [14], or gastro–oral transmission [15].

Mongolia is a landlocked country in Central East Asia, bordered by the Russian Federation in the north and People’s Republic of China in the south, west, and east. According to the age-standardized rate (ASR) of GC per 100,000 Mongolian population, Uvs Province (western part of Mongolia) has the highest (80.7) and Khentii Province (eastern part of Mongolia) has the lowest (11.2) incidence, with the country average of 32.4, which was ranked as the second highest globally [1].

The precancerous diseases of GC and its etiological risk factors including *H. pylori* infection are not well studied based on geographical location. In addition to high incidence and mortality rates of GC, digestive diseases have been constantly increasing in the last 10 years in Mongolia [2]. In this serious situation, defining etiologies of these diseases is urgently required, especially in areas with high incidence of GC. Therefore, this study aimed to examine the prevalence of *H. pylori* infection and other risk factors among dyspeptic patients in the major geographical locations (western, northern, southern, and central parts) in Mongolia.

## Methods

### Sampling, questionnaire and endoscopy of patients

We conducted a cross-sectional study among dyspeptic patients aged > 16 years, from 2014 to 2016. History of partial or total gastrectomy; treatment with bismuth-containing compounds, H2-receptor blockers, or proton pump inhibitors within 2 weeks prior to the start of the study and history of previous eradication therapy for *H. pylori* infection were the exclusion criteria.

We chose the places according to the geographical location and GC incidence rate in the 2012 Mongolian national data. The selected places were as follows: Ulaanbaatar City (central part of Mongolia, with an ASR of GC incidence of 31.3 per 100,000 population), Uvs Province (western; ASR, 80.7), Khuvsgul Province (northern; ASR, 37.0), Umnugovi Province (northern; ASR, 21.5), and Khentii Province (eastern; ASR, 11.5).

Age and gender were used as non-modifiable risk factors, and high salt intake and bad habits (tobacco smoking and alcohol drinking) were used as modifiable environmental risk factors. A previous nationwide study validated the questionnaire method by evaluating the excessive amount of salt using 24 h urine collection samples, revealing that the urine saline level was higher in daily salty tea drinkers than those who drink from other sources [16]. Therefore, to determine excessive amount of salt, we used the same questionnaire method to screen routine salty tea drinkers. For bad habits, tobacco smoking (daily smokers) and alcohol drinking (excessive amount of standard drink [350 mL glass of beer, 150 mL glass of 12% wine, or 44 mL glass of spirit] per monthly, weekly, or daily) statuses were used.

During endoscopic examination, the Los Angeles (LA) classification was used to determine gastroesophageal reflux disease [17]. Gastric mucosal atrophy was evaluated using the Kimura-Takemoto classification [18] and was classified as closed type (mild atrophy, limited in the antrum and lesser curvature of the proximal corpus) and open type (advanced atrophy, extended into the corpus and cardia). Three gastric mucosal tissues were taken from the antrum approximately 3 cm from the pyloric ring, which were used for rapid urease test (Mon-HP, developed at the Mongolian National University of Medical sciences, Ulaanbaatar, Mongolia), histological examination, and bacterial culture. Two more biopsies were taken from the corpus and incisura angularis (angulus) for histological examination. If ulcer or suspected cancer lesions were detected, additional biopsy specimen(s) was taken for histological diagnosis. Biopsy specimens for culture were immediately placed at − 20 °C on the day of endoscopic examination and stored at − 80 °C until used for culture testing. Blood samples from all participants were collected on the same day. The serum was separated and frozen at − 80 °C until analysis. Written informed consent was obtained from all participants, and the ethical permission was approved by the Mongolian Ministry of Health, Mongolian National University of Medical Sciences, and Oita University Faculty of Medicine (Yufu, Japan).

### Histological confirmation

All biopsy materials were fixed in 10% buffered formalin and embedded in paraffin. Serial sections were stained with hematoxylin–eosin and May-Giemsa stain. The stained slides were examined by a single pathologist (Tomohisa Uchida). The degree of chronic inflammation (mononuclear cell infiltration), acute inflammation (polymorphonuclear neutrophil infiltration), atrophy, intestinal metaplasia (IM), and bacterial density were classified into four grades, based on the updated Sydney system: 0, “normal”; 1, “mild”; 2, “moderate”; and 3, “marked” [19]. Samples with grade 1 or higher were considered positive.

### *Helicobacter pylori* infection status

*H. pylori* infection was positive based on culture or histology, confirmed by immunohistochemistry (IHC) or both rapid urease test (RUT) and serology that yielded positive results. For *H. pylori* culture, antral biopsy specimen was homogenized in normal saline solution and placed in a commercially available selective plate (Nissui Pharmaceutical Co. Ltd, Japan). The plates were incubated for up to 10 days at 37 °C under microaerophilic conditions (10% O_2_, 5% CO_2_, and 85% N_2_). *H. pylori* were identified based on colony morphology, Gram staining, and positive reactions for oxidase, catalase, and urease tests.

In histological examination, bacterial loads ≥ grade 1 on the updated Sydney system were considered positive for *H.* pylori [19]. IHC was also performed to confirm *H. pylori* infection as previously described [20]. Serology of *H. pylori* infection (anti-*H. pylori* IgG antibody) was evaluated using a commercially available ELISA kit (Eiken Co., Ltd., Tokyo, Japan) according to the manufacturer’s instructions.

### Statistical analysis

Discrete categorical variables were tested using Chi square test, and non-parametric continuous variables were calculated using Mann–Whitney test or Kruskal–Wallis test. A two-tailed *p* value of < 0.05 was considered statistically significant. Univariate and backward logistic regression analyses were used to calculate odds ratios. In the univariate analysis, *p *< 0.3 was included to calculate the multivariate backward logistic regression analysis. All statistical analyses were performed using IBM SPSS Version 22.0 (IBM Corp. Armonk, NY, USA) software.

## Results

### Patient sampling and demography

Endoscopy was performed on 905 dyspeptic patients from the capital city (Ulaanbaatar, n = 226; November 18–22, 2014), western (Uvs Province, n = 148; July 14–21, 2015), northern (Khuvsgul Province, n = 212; July 19–25, 2015), southern (Umnugovi Province, n = 176; August 4–8, 2016), and eastern (Khentii Province, n = 143; August 9–12, 2016) parts of Mongolia. All patients were examined by experienced Japanese and Mongolian endoscopists. A total of 169 patients were excluded from the study due to our exclusion criteria that patients were previously received *H. pylori* eradication. Finally, we included 736 dyspeptic patients. Among all patients, 68.2% (502/736) were females and 31.8% (234/736) were males, with the mean age ± SD of 43.6 ± 13.6 years, ranging from 15 to 87 years: 18.5% aged ≤ 30 years; 20.8%, 30–39 years; 24.9%, 40–49 years; 23.4%, 50–59 years; and 12.5%, ≥ 60 years. About 88.7% (653/736) belong to the major (Khalkh) and 11.3% (83/736) to the minor (non-Khalkh) ethnicity groups. The minor ethnicities were Bayad (44.6%, n = 37), Durvud (42.2%, n = 35), Khoton (7.2%, n = 6), Tuva (2.4%, n = 2), Darkhad (1.2%, n = 1), Khotgoid (1.2%, n = 1), and Myangad (1.2%, n = 1).

### Demographic and disease backgrounds by geographical location

Table 1 shows the demographic and endoscopic diagnosis based on geographical locations. Age and gender were not different based on the location, but ethnic groups were different. Most patients (66.7%) belonged to the minor ethnic (Bayad, Durvud, and Khoton) groups in the western part (Uvs Province) (*p *< 0.0001), whereas almost all patients in other locations belonged to the major (Khalkh) group. Northern and western parts had a significantly higher incidence of reflux esophagitis (LA grades, A–D) and atrophic gastritis (open type) compared with other regions. The prevalence of gastric and duodenal ulcer did not differ by location (Table 1). Endoscopic and histological diagnosis was not significantly different by ethnic group.

**Table 1:** Demography and diseases according to geography

Parameters	Geographical location					
	Eastern	Southern	Central	Northern	Western	*p* value
	(Khentii)	(Umnugovi)	Ulaanbaatar city	(Khuvsgul)	(Uvs)	
Number of cases	n = 115	n = 124	n = 206	n = 171	n = 120	
Mean age with SD	47.4 ± 12.6	44.3 ± 13.8	41.5 ± 15.4	43.5 ± 12.7	42.6 ± 11.9	NS
Age range	17–87	15–79	17–79	19–75	16–74	NS
Gender						
Female	73%	71%	65.5%	65.5%	69.2%	NS
Male	27%	29%	34.5%	34.5%	30.8%	NS
Ethnicity						
Major	97.8%	100%	N/A^a^	98.4%	33.3%	0.0001
Minor	2.2%	0%		1.6%	66.7%	
Endoscopy diagnosis						
Gastric ulcer	3.5%	3%	2.4%	2.9%	2.5%	NS
Duodenal ulcer	0%	0.8%	1.9%	1.8%	1.7%	NS
Erosive GERD	5.2%	9.7%	5.3%	11.7%	14.2%	0.03
Open type atrophy	1.3%	1.8%	1.4%	6.0%	13%	0.0001
Histology diagnosis						
Intestinal metaplasia	19.1%	21.8%	16%	23.4%	32.5%	0.01

^a^ We could not obtain data

### *Helicobacter pylori* infection rate by demography

Table 2 shows the different diagnostic tests for *H. pylori* infection based on age group, gender, and geographical locations of all dyspeptic patients. Based on our criteria (positive by culture or histology, or positive for both RUT and serology), the prevalence of current *H. pylori* infection was 80.0% (589/736), with 5.2% (38/736) being positive by serology only and therefore were regarded as the cases with past infection. The remaining 14.8% (109/736) were regarded as the uninfected group. Based on gender, the prevalence of current infection was not significantly different from 78.2% (183/234) in men and 80.9% (406/502) in women. The infection rate based on age group was 83.8% for patients < 30 years, 88.2% for 30–39 years, 81.4% for 40–49 years, 74.4% for 50–59 years, and 68.5% for ≥ 60 years. *H. pylori* infection peaked in patients aged 30–39 years, but was lower in older patients (*p *= 0.005).

**Table 2:** *H. pylori* infection by different tests and overall infection status

Parameters	*H. pylori* different tests						*H. pylori* diagnosis			*p* value
	N	RUT (%)	Culture (%)	Histology (%)	IHC (%)	Serology (%)	Current (%)	Past (%)	Uninfected (%)	
Overall	736	67.3	48.2	75.8	76.0	64.3	80.0	5.2	14.8	
Age group										
< 30	136	73.5	55.1	79.4	79.4	69.9	83.8	3.7	12.5	0.005
30–39	153	79.7	52.3	83.7	83.7	75.2	88.2	5.2	6.6	
40–49	183	62.8	43.2	76.0	76.5	59.0	81.4	4.9	13.7	
50–59	172	61.6	45.3	70.3	70.3	62.2	74.4	5.2	20.3	
< 60	92	56.5	46.7	67.4	67.4	52.2	68.5	7.6	23.9	
Gender										
Female	502	67.9	51.0	77.1	77.1	66.9	80.9	5.0	14.1	NS
Male	234	65.8	42.3	73.1	73.5	58.5	78.2	5.6	16.2	
Place										
Umnugovi	124	68.5	63.7	83.9	83.9	70.2	87.1	1.6	11.3	0.03
Khentii	115	68.7	80.0	80.9	80.9	72.2	87.0	4.3	8.7	
Khuvsgul	171	62.6	19.3	74.9	74.9	63.7	78.4	8.2	13.5	
Ulaanbaatar	206	67.0	63.6	71.8	72.3	58.7	74.8	5.3	19.9	
Uvs	120	71.7	16.7	70.8	70.8	60.8	77.5	5.0	17.5	

*RUT* rapid urease test, *IHC* immunohistochemistry

The infection rate based on geographical locations was highest in Umnugovi (87.1%; 108/124), followed by Khentii (87%; 100/115), Khuvsgul (78.4%; 134/171), and Uvs (77.5%; 93/120) provinces and Ulaanbaatar city (74.8%; 154/206) (*p *= 0.03). The prevalence of *H. pylori* was not different based on ethnic groups: 80.1% (523/653) in the major and 79.5% (66/83) in the minor ethnic groups (*p *= 0.5).

### Histological diagnosis based on *H. pylori* infection

**Fig. 1:**
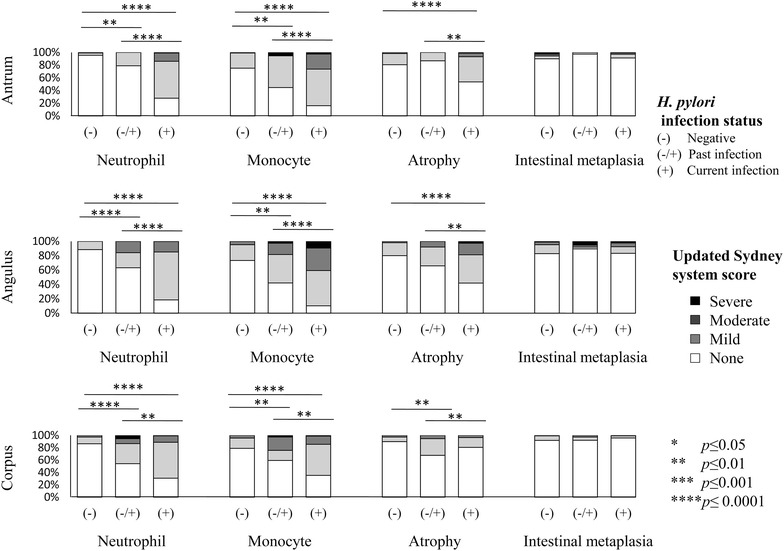
Updated Sydney system scores by *H. pylori* infection status and different gastric biopsy sites

Figure 1 shows the histological diagnosis based on the updated Sydney system by *H. pylori* infection. Acute (neutrophil) and chronic inflammation (monocyte) scores were significantly higher in patients with current *H. pylori* infection compared to those with past infection and those who were uninfected based on each biopsy site (the antrum, angulus, and corpus). Acute and chronic inflammation scores were also significantly higher in those with past infection than in the uninfected group in each biopsy site. The atrophy score was also significantly higher in patients with current infection status than those with negative and past infection status in the antrum and angulus. The corpus atrophy scores were higher in the past infection group than in the current and uninfected groups. Interestingly, IM scores in all sites were independent of *H. pylori* infection (Fig. 1).

### *Helicobacter pylori* infection rate by endoscopy and histological diagnosis

**Fig. 2:**
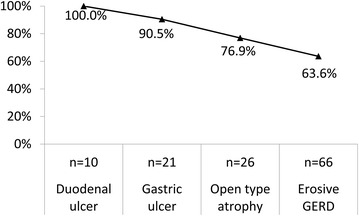
The prevalence of *H. pylori* infection by endoscopic diagnoses

Figure 2 shows the *H. pylori* infection rate according to endoscopic diagnoses. On esophageal endoscopy, 63.6% (42/66) of overall erosive esophagitis (LA grade, A–D) patients were infected with *H. pylori*; in the stomach, 90.5% (19/21) of gastric ulcer and 76.9% (20/26) of open type atrophy; and in the duodenum, 100% (10/10) of duodenal ulcer patients were infected with *H. pylori*.

Next, we summarized *H. pylori* infection status based on the overall histological diagnosis as “yes” or “no” status for the following groups: normal “yes” status, all scores in three sites were 0 (n = 57); gastritis “yes” status, neutrophil or monocyte scores were 1–3 in at least one site (n = 659); atrophy “yes” status, atrophy score was 1–3 in at least one site (n = 483); and IM “yes” status, IM score was 1–3 in at least one site (n = 160) (Table 3).

**Table 3:** *H. pylori* infection status according to histological diagnosis

Parameters	Case number	Current infection (%)	Past infection (%)	Uninfected (%)	*p* value
Histological diagnosis					
Normal					
No	679	85.6	4.4	10.0	0.0001
Yes	57	14.0	14.0	72	
Gastritis					
No	77	11.7	11.7	76.6	0.0001
Yes	659	88.0	4.4	7.6	
Atrophy					
No	253	63.2	8.3	28.5	0.0001
Yes	483	88.8	3.5	7.7	
IM					
No	576	79.9	5.7	14.4	NS
Yes	160	80.6	3.1	16.3	

*IM* intestinal metaplasia

As expected, the prevalence of *H. pylori* infection was significantly lower in the normal group (*p* < 0.0001), but significantly higher in the gastritis (*p* < 0.0001) and atrophy (*p* < 0.0001) groups. Importantly, the presence of IM was independent of *H. pylori* infection.

### *Helicobacter pylori* infection and other risk factors in high-risk diseases for GC

**Fig. 3:**
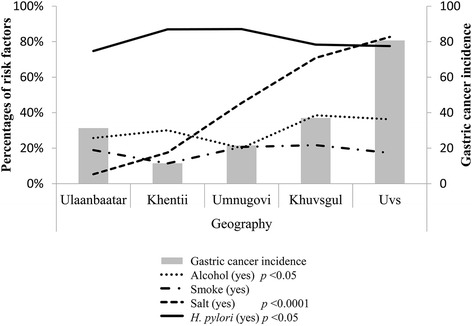
The prevalence of *H. pylori* infection and other environmental factors by geographical location

Since IM was diagnosed among uninfected groups with gastritis, environmental factors were investigated in detail. Figure 3 shows the prevalence of *H. pylori* infection and other environmental factors by geographical location. Kruskal–Wallis test showed excessive amount of salt usage and alcohol drinking status were significantly higher in western and northern parts of Mongolia (Fig. 3).

Next, we evaluated these factors according to the high-risk disease group (erosive GERD for cardia GC and atrophy and IM for non-cardia GC). Table 4 shows the univariate and multivariate analysis results.

**Table 4:** *H. pylori* infection and other risk factor status according to high-risk diseases for GC

Parameters	Erosive GERD		Atrophy		Intestinal metaplasia	
	*p* value	OR (95% CI)	*p* value	OR (95% CI)	*p* value	OR (95% CI)
Univariate						
*H. pylori* (yes)	0.0001	0.4 (0.2–0.6)	0.0001	4.6 (3.1–6.7)	0.6	0.9 (0.5–1.4)
Age group (> 40)	0.002	2.3 (1.4–4.8)	0.9	1.0 (0.7–1.4)	0.0001	3.4 (2.2–5.2)
Gender (male)	0.0001	3.5 (2.1–5.9)	0.4	0.8 (0.6–1.2)	0.04	1.5 (1.0–2.1)
Salt (yes)	0.01	1.9 (1.1–3.3)	0.1	0.6 (0.4–0.8)	0.06	1.4 (1.0–2.0)
Smoke (yes)	0.02	2.0 (1.1–3.6)	0.6	1.1 (0.7–1.6)	0.1	1.4 (0.9–2.2)
Alcohol (yes)	0.5	1.2 (0.7–2.0)	0.6	1.0 (0.7–1.5)	0.1	1.3 (0.9–2.0)
Multivariate						
*H. pylori* (yes)	0.004	0.4 (0.2–0.7)	0.0001	5.1 (3.5–7.7)	–	
Age group (> 40)	0.002	2.9 (1.4–5.6)	–		0.0001	3.8 (2.4–5.9)
Gender (male)	0.0001	3.3 (1.9–5.8)	–		–	
Salt (yes)	0.007	2.1 (1.2–3.7)	–		0.04	1.5 (1.0–2.2)
Smoke (yes)	–		–		–	
Alcohol (yes)	–		–		–	

*OR (95% CI)* odds ratio (95% confidence interval)

Multivariate logistic regression analysis showed that *H. pylori* infection was associated with increased risk for corpus gastritis (odds ratio, 54.0 [95% confidence interval 25.8–113.2]); *p *< 0.0001). *H. pylori* infection was associated with increased risk for atrophy and decreased risk for erosive GERD. Our finding supports the consensus that *H. pylori* infection is a protective factor against GERD [21]. Age > 40 years, male gender, and excessive amount of salt intake increased the risk of erosive GERD, and age > 40 years (3.8 [2.4–5.9]; *p *< 0.0001) and excessive salt intake (1.5 [1.0–2.2]; *p *< 0.02) increased the risk of IM.

## Discussion

Mongolia is known for the highest mortality from GC in the world [1] and upper digestive tracts diseases are common [2]. Therefore we characterized features of gastric precancerous diseases and ulcerative diseases as well as demonstrating its risk factors among the Mongolian population. Gastric atrophy and IM are considered as the precursor disease for non-cardia GC [21] while GERD is considered as precursor disease for cardia GC [22]. We found that all these precursor diseases were higher in western followed by northern, southern, central and eastern part of Mongolia whereas ulcerative diseases were not significantly different by geographical location.

*H. pylori* infection is the major etiological factor that causes atrophic gastritis and development of IM is considered the most important recognizable risk factors for GC [21]. We found that *H. pylori* infection was associated with precursor GC diseases and ulcerative diseases among Mongolian patients. Most patients with atrophy and IM were infected with *H. pylori* (Table 3). For PUD, it was similar with other studies that approximately 90% of duodenal ulcer and 80% of gastric ulcer were associated with *H. pylori* infection [23]. PUD and GC can lead to fatal conditions, such as severe massive hemorrhage, perforation, and peritonitis [24]. For precancerous disease, multivariate analysis showed that *H. pylori* infection was associated with gastritis and atrophy (Table 4). Permanent *H. pylori* infection can cause chronic active gastritis, resulting in loss of glands (atrophy) and then development of IM [25]. *H. pylori* infection causes nearly 90% of non-cardia GC, and the most GC incidences were high in East Asian countries [1]. In China, Japan, and Korea, prospective studies confirmed that *H. pylori* infection played a major role in the occurrence of gastric atrophy and IM, which are the precancerous conditions, especially for non-cardia GC [26–28]. Indirect evidence from populations with a very low prevalence of *H. pylori,* such as the Malays of north eastern Peninsular Malaysia, is consistent with the idea that *H. pylori* decreased the risk of developing pre-neoplastic lesions and GC [29]. Since *H. pylori* infection is treatable, a 30–40% reduction in the incidence of GC was observed among treated subjects [30], and the cure of *H. pylori* infection reduced the precancerous disease progression to GC [31]. Therefore, *H. pylori* infection should be treated to reduce GC incidence among the Mongolian population. A previous study reported that *H. pylori* infection in adults mainly occurred before 15 years old [32]. Our study showed that the peak age of *H. pylori* infection was 30–39 years, while it was lower in older age (Table 2). Oral–oral and fecal–oral routes were considered as the crucial routes of *H. pylori* infection transmission [33]. Further risk factor studies are required to determine the transmission routes. The prevalence of *H. pylori* infection was generally high in Mongolia and did not differ by geographical location.

Interestingly, in spite of *H. pylori* infection, excessive amount of salt intake was dramatically higher in the northern and western parts of Mongolia, where precancerous diseases, such as erosive esophagitis (for cardia cancer), severe atrophy, and IM (for non-cardia cancer), were highly prevalent (Table 1). These results were consisted with the GC incidence (Fig. 3). *H. pylori* infection together with high salt intake has been reported to induce more gastric damage, such as atrophy and IM [34]. Furthermore, a prospective study confirmed that even salt alone could induce gastric atrophy and IM [35]. High salt intake causes damage to the gastric epithelium, followed by rapid restoration [36] and cell replication [37, 38]. Salting of foods increases the mutagenicity of nitrosated foods [39], and salt has been experimentally proven to increase the permeability of the experimental rat’s gastric mucosa to carcinogenic agents [40, 41]. *H. pylori*-related gastric atrophy is considered as the major precursor disease for IM [42]; however, IM may also develop because of other harmful environmental factors, because of its gastric tissue repairing process [43, 44]. Our study showed that excessive amount of salt intake and advanced age (> 40) increased the risk of IM (Table 4). Moreover, salt, advanced age, and male gender increased the risk of GERD. Some previous studies have found that increased salt consumption was associated with GERD, which was attributed to the delayed gastric emptying and increased pancreaticobiliary secretion after high salt intake [45, 46]. *H. pylori* infection, especially with a strain bearing CagA, is commonly considered a protective factor against GERD [47]. Our study results are consistent with the concept that *H. pylori* reduced the risk of erosive GERD. Excessive amount of salt intake seemed to play a major role for esophageal and gastric diseases, especially in western and northern parts of Mongolia. Further prospective studies are required to determine the pathogenesis of these diseases.

## Conclusions

Based on our current result, we concluded that *H. pylori* infection is a major health problem for stomach diseases, such as PUD and atrophic gastritis among the Mongolian population. Nationwide screening and eradication of *H. pylori* infection are required. In addition, managing modifiable risk factors such as reducing excessive amount of salt intake and bad habits might help in decreasing erosive GERD and IM.
